# Physician and Pharmacist Medication Decision-Making in the Time of Electronic Health Records: Mixed-Methods Study 

**DOI:** 10.2196/humanfactors.9891

**Published:** 2018-09-25

**Authors:** Kathryn Mercer, Catherine Burns, Lisa Guirguis, Jessie Chin, Maman Joyce Dogba, Lisa Dolovich, Line Guénette, Laurie Jenkins, France Légaré, Annette McKinnon, Josephine McMurray, Khrystine Waked, Kelly A Grindrod

**Affiliations:** 1 School of Pharmacy University of Waterloo Waterloo, ON Canada; 2 Centre for Bioengineering and Biotechnology University of Waterloo Waterloo, ON Canada; 3 Systems Design Engineering Faculty of Engineering University of Waterloo Waterloo, ON Canada; 4 Faculty of Pharmacy and Pharmaceutical Sciences University of Alberta Edmonton, AB Canada; 5 Biomedical and Health Information Sciences University of Illinois at Chicago Chicago, IL United States; 6 Faculty of Medicine Université Laval Ville de Québec, QC Canada; 7 Faculty of Pharmacy University of Toronto Toronto, ON Canada; 8 Population Health and Optimal Health Practices, CHU de Québec Research Centre Université Laval Ville de Québec, QC Canada; 9 Faculté de pharmacie Université Laval Ville de Québec, QC Canada; 10 Healthcare Navigators Toronto, ON Canada; 11 Patient Partner Toronto, ON Canada; 12 Lazaridis School of Business and Economics Wilfrid Laurier University Waterloo, ON Canada

**Keywords:** shared decision-making, electronic health records, collaboration, interprofessional collaboration, medication management

## Abstract

**Background:**

Primary care needs to be patient-centered, integrated, and interprofessional to help patients with complex needs manage the burden of medication-related problems. Considering the growing problem of polypharmacy, increasing attention has been paid to how and when medication-related decisions should be coordinated across multidisciplinary care teams. Improved knowledge on how integrated electronic health records (EHRs) can support interprofessional shared decision-making for medication therapy management is necessary to continue improving patient care.

**Objective:**

The objective of our study was to examine how physicians and pharmacists understand and communicate patient-focused medication information with each other and how this knowledge can influence the design of EHRs.

**Methods:**

This study is part of a broader cross-Canada study between patients and health care providers around how medication-related decisions are made and communicated. We visited community pharmacies, team-based primary care clinics, and independent-practice family physician clinics throughout Ontario, Nova Scotia, Alberta, and Quebec. Research assistants conducted semistructured interviews with physicians and pharmacists. A modified version of the Multidisciplinary Framework Method was used to analyze the data.

**Results:**

We collected data from 19 pharmacies and 9 medical clinics and identified 6 main themes from 34 health care professionals. First, Interprofessional Shared Decision-Making was not occurring and clinicians made decisions based on their understanding of the patient. Physicians and pharmacists reported indirect Communication, incomplete Information specifically missing insight into indication and adherence, and misaligned Processes of Care that were further compounded by EHRs that are not designed to facilitate collaboration. Scope of Practice examined professional and workplace boundaries for pharmacists and physicians that were internally and externally imposed. Physicians decided on the degree of the Physician-Pharmacist Relationship, often predicated by colocation.

**Conclusions:**

We observed limited communication and collaboration between primary care providers and pharmacists when managing medications. Pharmacists were missing key information around reason for use, and physicians required accurate information around adherence. EHRs are a potential tool to help clinicians communicate information to resolve this issue. EHRs need to be designed to facilitate interprofessional medication management so that pharmacists and physicians can move beyond task-based work toward a collaborative approach.

## Introduction

In clinical settings, medication-related decisions are often passed verbally among patients, doctors, nurses, and pharmacists, and the message can become distorted. Too often, however, critical information is not shared, even when an electronic health record (EHR) is used, and the decision to prescribe or not prescribe, to take or not take a medication, is made with missing or distorted information [1-4]. Health systems now promote an ethos of partnership where providers and patients navigate complex relationships and interactions. The shift from a patient-physician decision-making dyad to a network of providers introduces more complexity into what are often byzantine processes that precede health decisions. Nevertheless, patients often rely on a trusted health care professional’s (HCP’s) expertise to make important decisions where the situation is emergent or ambiguous (eg, having a surgery or starting a new medication) [5,6]. Research has not yet empirically characterized how current communication between health care practitioners affects care, and specifically how EHRs can strengthen communication by making information easier to access [7].

A medication-related decision involves, at minimum, a patient, a prescriber, and a pharmacist, and all parties are engaged in a process of shared decision-making (SDM) [8,9]. SDM is based on a model of communication where HCPs and patients both contribute to clinical decisions in unique ways [10,11]. The HCPs share information about the benefits and risks of different treatment options; the patients describe their preferences and values as they relate to their treatment options. Interprofessional shared decision-making (IP-SDM) involves multiple HCPs and is emerging as a response to care increasingly being delivered by interprofessional teams to collaboratively work with a patient to decide on the best course of action [12]. A systematic review of the adoption of SDM by HCPs concluded that while it is unclear whether interventions that promote the adoption of SDM are effective, interventions that target patients and HCPs simultaneously are more effective than ones that only target one group [13]. The evolution of IP-SDM is challenging our beliefs about how and when HCPs actively communicate with each other and with patients as well as about the role EHRs may play in decision-making.

Adverse drug events (ADEs) are one of the outcomes of miscommunication in the medication management process. The costs of ADEs to the health care system are staggering, yet in one US study, physician reviewers determined that of the 30% inpatients who experienced ADEs, the events were preventable in 44% cases [14-16]. While these medication-related problems are the symptom of a complex and disconnect health care system, the inclusion of pharmacists in medication management has reduced the rates of ADEs as well as health care costs [17]. ADEs account for somewhere between 1.4% and 15.4% of hospital admissions in the United States and Canada, accounting for an estimated 177,504 emergency department visits by US patients aged ≥65 years and increasing the mean length of hospital stay from 8 to 20 days [18-20]. SDM is known to improve communication, lessen ADEs, and, overall, lower health care costs [21,22]. Through greater communication and collaboration between HCPs and patients, IP-SDM provides a platform that has significant potential to further lessen ADEs and to continue lower health care costs [23].

In most health care settings, pharmacists and physicians often do not communicate well because they largely work *independently and in parallel* with each other, rather than collaboratively [24]. Furthermore, there can be challenges in communication due to differences of opinion on role, reluctance to challenge, different work schedules, and different information priorities [25-27]. For example, how physicians and pharmacists communicate and make decisions with each other is based on perceptions about the role each one plays in a person’s care and is tightly tied to ideas about pharmacists’ scope of practice. According to Nugus et al, there is a clear acknowledgment in health care that physicians are the ones with “formal responsibility of patient care” and that they are omnipresent in care [28]. As a result, EHRs may reflect the physician’s information or decision-making needs more than those of the pharmacist or the patient. The challenge in designing interdisciplinary EHRs is that they need to account for the workflow and communication models of different professions. It is important that physicians and pharmacists have a good communication because it is essential to go beyond transactional interactions to ensure optimal therapeutic outcomes for patients [29]. This research has been conducted to better foundationally understand how pharmacists and physicians communicate, which can be used to lessen medication-related errors, lower health care costs, and design and improve EHRs that facilitate collaboration.

The objective of this exploratory study is to examine how physicians and pharmacists understand and communicate patient-focused medication information with each other and to identify barriers to IP-SDM for medication management that should inform designing EHRs that support IP-SDM. This research will allow for the design and refinement of EHRs that can be designed to facilitate better communication, improve medication management, and ultimately contribute to improved care.

## Methods

### Research Design

This research was part of a larger mixed methods study on SDM in the context of EHRs that included observations; interviews; and think-aloud discussions with patients, primary care physicians, and pharmacists. This paper focuses on qualitative, semistructured interviews with physicians and pharmacists. We have taken a pragmatic stance, recognizing that a constructivist view of the truth can be tempered with the need to conduct research that informs health care decision-making [30]. Our analysis was guided by a framework analysis method that provides both a systemic and flexible approach to multidisciplinary data analysis [31].

We conducted interviews in community pharmacies and primary care clinics across Canada using provinces to represent different levels of primary care integration and adoption of EHRs (Table 1). This research received ethics approvals from the University of Waterloo, the University of Alberta, Wilfrid Laurier University, Université Laval, the University of Toronto, and Dalhousie University.

### Recruitment and Participants

The research team used a purposive sampling approach to identify a broad spectrum of practice sites. Recruitment was conducted through several venues including posters, social media, and snowball sampling from previous and existing contacts of the research team. We included pharmacists and family physicians practicing in Ontario, Alberta, Quebec, and Nova Scotia.

### Data Collection

Three research assistants conducted and audiorecorded the interviews. One of the research assistants was a PhD candidate and experienced qualitative researcher (KM) and the other two were PharmD students (KW, JB). The three interviewers jointly conducted 3 interviews to train the student research assistants in semistructured interview techniques, and they regularly met throughout the data collection period to compare interview notes and transcripts. All three research assistants interviewed participants in Ontario, with KW completing all of the interviews in Quebec and Alberta and JB completing all of the interviews in Nova Scotia. Field notes recorded during and after the interviews documented the environment, external influencers or distractions, and participants; specific questions were added to better understand the decision-making approach.

**Table 1:** Description of in-place electronic health records (EHRs) and primary care models in Alberta, Ontario, Quebec, and Nova Scotia between December 2015 and October 2016.

Health record		Province			
		Alberta	Nova Scotia	Ontario	Quebec
**EHR^a^**		Netcare	SHARE^b^	ClinicalConnect^c^	Dossier Santé Québec
	Medication profile	Yes	Drug Information System	Only hospital medications	Yes
	Laboratory values	Yes	Yes	Yes	Yes
	Medical imaging	Yes	Yes	Yes	Yes
	Integrated systems	Pharmaceutical Information Network	Drug Information System	Ontario Laboratories Information System	N/A^d^
	Other information	Hospital visits, surgeries, drug alerts, allergies or intolerances, immunizations	Hospital admissions or discharge information, history and consulting notes	Allergies, medical reports, pathology and microbiology results	Electronic prescriptions
Physician access to EHR		Yes	Yes	Yes	Yes
Pharmacist access to EHR		Yes	Drug Information System^e^	No	Yes
Team-based health care		Primary Care Network health care teams	Collaborative care teams	Family health teams	Family medicine groups
Pharmacist integration in team-based health care		Yes	Yes	Yes	Government promotes close ties between community pharmacies and family medicine groups

^a^Information collected in this table reflects health care at the time of the interviews and may have changed since.

^b^SHARE: Secure Health Access Record.

^c^South Western ON. EHRs are region specific in Ontario; separated into 3 regions.

^d^N/A: not applicable.

^e^Access to laboratory values in near future.

Interviews with HCPs consisted of two parts: (1) medication-focused decision-making and (2) interviewee’s opinion of EHRs. HCPs were interviewed where they practiced, either in the pharmacy or the physician’s office. Interviews focused on how the pharmacist or physician presented information to patients; how collaboration was approached during care, specifically in relation to medication prescribing or problem solving; how they interacted with EHRs or electronic medical records (EMRs) used in their practice; and finally potential areas for developing new EHRs. The interview guide is available in Multimedia Appendix 1.

### Data Analysis

We employed a modified version of the *Multidisciplinary Framework Method* to analyze the data [24]. A multidisciplinary team, including engineers, clinicians, health researchers, business and communication researchers, patients, and a patient navigator, was involved in data analysis. The steps followed were as follows: (1) interviews were transcribed verbatim; (2) core research team members read the transcripts and listened to the audiorecordings to familiarize themselves with the interviews; (3) core team members thematically coded the data; (4) the entire team thematically coded a subset of 5 interviews; (5) the team codes were used to develop a working analytic framework; (6) 2 team members (KM, KW) recoded the data; and (7) finally, the data were presented to the entire team for discussion and refinement. Data were stored, organized, and reported using QSR NVIVO 11 Software (QSR International Pty Ltd. Version 11, 2017). Any names and identifiers were made anonymous in the transcription process. Multiple triangulation of the data was achieved using a variety of geographic sources, multiple coders, and a multidisciplinary team of researchers interpreting the results [32].

## Results

### Study Population

In total, we interviewed 25 pharmacists and 9 family physicians (Table 2). On average, the HCPs had been with their current clinic for 8 years and had been practicing for 15 years. Compared with physicians, a larger sample of pharmacists was recruited to account for variability in practice setting; the latter included pharmacists who worked in chain pharmacies (n=5), independently owned pharmacies (n=12), and team-based medical clinics (n=4).

### Thematic Analysis

Initial coding conducted by the core research team led to the identification of 46 codes, which were then developed into 5 themes describing the different elements of how pharmacists and physicians make medication-related decisions with patients: *workflow, communication, accuracy, decision-making*, and *computer systems*.

**Table 2:** Participant demographics collected at the time of interview (N=34).

Characteristics		Family physicians	Pharmacists
**Province, n**			
	Nova Scotia	0	4
	Quebec	2	2
	Ontario	6	15
	Alberta	1	4
Total participants, n		9	25
Team environment, n		9	4
Independent practice, n		0	21
Years in practice^a^		12.6	16.2
Average duration of current practice (years)		9.9	7.1
Average age of participants (years)^a^		43.4	39.8
**Participant age group (years), n** ^a^			
	25-35	2	7
	36-45	4	12
	46-55	2	4
	55+	1	2
**Gender**			
	Male	4	11
	Female	7	14

^a^Information regarding age and years in practice was not collected from 1 family physician participant.

As part of the multidisciplinary framework, we held a 2-day research meeting where the entire multidisciplinary team participated in the analysis. Research group members came to the meeting having individually coded the same 5 interviews. Through a process of negotiation, individual codes were rearranged into 81 subthemes and 6 major themes as outlined below (Table 3). KM and KW recoded the remaining interviews using the new framework, with no additional themes arising. The new coding framework placed a more significant focus on how pharmacist-physician relationships and scopes of practice affect medication-related decisions (Table 3). We found that decision-making was influenced by the information, processes, and communication factors related to EHRs, which, in turn, were influenced by the physician-pharmacist relationships and scopes of practice.

**Table 3:** Themes related to interprofessional medication-related decision-making between physicians and pharmacists.

Theme	Subthemes	Description
Interprofessional shared decision-making (IP-SDM)	IP-SDM intentions Decision point Making the decision Assumptions about patients Patient communication IP-SDM	Pharmacists and physicians did not describe IP-SDM in their practices and acted as unintentional gatekeepers to medication information. Professionals make decisions based on their individual understanding of the patient’s situation and educate the patient based on that decision.
Communication between physicians and pharmacists	Reasons for health care professionals (HCPs) to communicate with patients Reasons for HCPs to communicate with each other Flow of information Communication workarounds Method of communication Availability How to document in the medical or pharmacy chart Risk communication Patient as messenger	Pharmacists and physicians often communicate with each other indirectly through patients, faxes, or receptionists. Yet, both groups are cautious about the expansion of electronic health records (EHRs) and how EHRs influence their ability to work.
Information exchange between physicians and pharmacists	Important information for patient care Information detectives Data collection and entry Multiple users Place of access Context of data entry Adherence Information scarcity limits roles Design features Timeliness	Pharmacists and physicians require information not accessible through current Web-based health platforms to provide patient care. Even in situations where the information was available, it was clear that relationships drove information sharing. Most critically, physicians required access to information about medication adherence, while pharmacists required clear access to medication indications.
Process of care	System design (fill and bill) Identifying patients in need of care Stages of care Technology limits practice Decision-making Workarounds Documentation of process Workflow bottlenecks Prioritization	Pharmacists and physicians find that current systems do not typically align with their decision-making processes and do not support collaboration in daily workflow.
Scope of practice	Responsibility to diagnose Negotiating role boundaries Accountability Medication management Mentorship and role modeling Monitoring	The workplace and professional boundaries for pharmacists and physicians are both internally and externally imposed. This includes how each group negotiates the boundaries of its job, how each group negotiates its interactions with each other and with patients, and how relationships, or lack thereof, impact the ability of each group to carry out its roles and responsibilities.
Physician-pharmacist relationship	Physical distance Community versus primary care pharmacist 5 Ws of shared understanding Filling the gap or tailoring Building collaborative work environments Transactional communication	Relationships were strongly influenced by physician location, nature of the task, and a power imbalance.

### Interprofessional Decision-Making

In the interviews, we asked about how different treatment options were presented, how patients’ values were taken into account, and whether the participant knew about IP-SDM. We observed that IP-SDM was not an active part of the typical decision-making process. Rather, we identified a spectrum of decision-making, where the most common approaches to decision-making included paternalism and informed decision-making, as outlined below, rather than IP-SDM.

In the paternalistic decisions that were both described and witnessed, the physician or pharmacist made a decision because they “assumed,” “understood,” or “knew” it was the “best”, and then, they “informed” the patient regarding what the patient should do. In other words, the physicians or pharmacists “shared” *their* final decision rather than sharing the decision-making process:

I really do consider also the patient's preference or pre-knowledge or understanding. Have I considered all the factors; the patient factors, cost factors? That kind of thing I try to make it so it's sort of like rational prescribing, thinking is there a reason to give it to them?

Physician 1205, Family Health Team, Ontario

During informed decision-making, pharmacists and physicians focus on educating patients well enough to allow them to make a decision. The goal is to offer recommendations, help the patient understand why the HCP offered the recommendation, and allow the patient to choose whether he or she wants to pursue the recommended course of action:

I want them to make an informed decision. I want them to understand what's going on with their health. I want them to understand what the options are and why we're pursuing those options. I want them to make an informed decision about whether they want to move forward with a particular treatment course or not and understand the rationale for that.

Physician 1202, Family Health Team, Ontario

One of the challenges of informed decision-making is that the information could “scare” the patient. It is unrealistic for all patients to become as well educated as an HCP about a medical decision:

I don’t want to give more information than necessary, especially if I see that a patient is more anxious during the beginning of the counselling, and even more so if the patient doesn't want to take the medication or is scared to take the medication.

Pharmacist 1121, Quebec, Independent Pharmacy

Pharmacists who worked in teams talked of making decisions *with* physicians rather than patients:

It was last Wednesday, was the last day that I worked there, and it was more I help the physician choose the medication. Not so much the patient themselves. It was a very complex case and the physician had asked me to meet with the patient first to do a medication review appointment.

Pharmacist 1124, Family Health Team Ontario

### Communication Between Pharmacists and Physicians

Communication between pharmacists and physicians is heavily dependent on the fax machine. Unlike a phone, faxed documents provide a written record of an encounter. However, fax machines are not connected with pharmacist and physician information systems, reducing the efficiency of their use.

We almost prefer a fax than phone a physician. We phone if it's an immediate thing, but faxing gives us, again, the detailed paper, dated and detailed work that we can keep track of. That's what we try to do

Pharmacist 1109, Independent Clinic, Nova Scotia

A common complaint among participants was that the standard processes to request information from another HCP are flawed. Pharmacists felt that they were limited by having to wait for a reply to a fax, and physicians often mentioned waiting until they had time to track down a pharmacist they trusted. The notion of a centralized way to communicate information was met with positive reactions. Being able to access key information without actively and asynchronously communicating with another HCP was identified as a way to streamline the sharing of basic medical information (eg, diagnosis, prescriptions, and lab results). Communication might then be focused around sharing meaningful information, such as patient histories or complex care regimens. Participants were concerned that information is not properly being communicated and may be missing or incorrectly documented. Pharmacists reported rarely being able to get past gatekeepers, such as office staff.

There's the ward clerk who won't let you through to the doctor. It's really difficult to get a doctor on the phone unless if they're calling you

Pharmacist 1102, Independent Pharmacy, Ontario

At instances where pharmacists are colocated with physicians, face-to-face interactions have the potential to foster the development of a trusting relationship. However, even when pharmacists and physicians are colocated, pharmacists still negotiate the power differential by modulating communication.

I don't go to a physician and say, “You must do this.” I say, “This is the problem that this patient is having on these medications. Here are our options. The options are A, B, and C. I like A because this, this, and this. I like B because of this, this, and this. What do you think we should do?” I never try and tell them what to do.

Pharmacist 1125, Family Health Team, Ontario

### Information Exchange Between Pharmacists and Physicians

Pharmacists and physicians use different pieces of information to provide patient care. Physicians record diagnostic information, including physical evaluations and tests, while pharmacists keep detailed records of medications provided. Most of the interviewed community pharmacists did not have access to the reason a medication was prescribed, the diagnostic test, or laboratory results. They assessed appropriateness and dispensed medications using the limited information contained on a prescription or patient recall. Additional or clarifying information needed to be requested from the physician. Even in situations where pharmacists had access to information through an EHR, issues related to missing information, and the consequent need to contact a physician to gain access to it, were mentioned.

Maybe there's some piece of information that we're missing and that's where you ask questions. If they're asking for refills too soon then it may be, “Why are you needing this more than what has been prescribed? Are you taking more than what was on the instructions that we have? Has someone told you to take more?”

Pharmacist 1124, Family Health Team, Ontario

Pharmacists often were missing information on the reason medications were prescribed. Not knowing why or how a prescriber decided on a medication not only limited pharmacists’ ability to properly educate patients about their medications but also limited their ability to participate in decisions to start, change, or stop a medication.

I would just say that getting information on the indication would be one. Trying to find out what they're taking the medication for and what they're hoping it's going to do for them would be two of the first questions.

Pharmacist 1107, Nova Scotia, Independent Practice

Physicians were also concerned that pharmacists do not have sufficient patient information to effectively prescribe, deprescribe, or assess a patient’s medications. Physicians were missing information about how a medication is taken after it was prescribed. Occasionally, there were plans in place to confirm prescription pick up and adherence, but the absence of adherence data was a clear gap in information. Both groups cited that the benefit of an EHR was enabling improved communication and overall patient care.

We've got a system [to help us keep track of] adherence. It's a really difficult point, and it's a really important point that I think we need to look because it's not good right now.

Physician 1201, Ontario, Family Health Team

### Process of Care

Pharmacists and physicians follow different processes for providing care, which are reflected in different information systems used in their daily workflow. Physicians use clinical data from physical assessment, lab values, and diagnostic imaging to make treatment decisions. Their office-based EMRs support documentation of their patient encounters, assessments of the information, and prescribing history. In community pharmacies, pharmacy practice management systems support dispensing and provide patient information sheets, auxiliary warning labels, warnings about adherence, and drug interaction alerts. Even in Alberta and Quebec, where there are province-wide EHRs that include lab values and dispensing information, the dispensing information is not integrated into the physician’s EMR and the clinical information is not incorporated into the pharmacist’s pharmacy practice management systems.

My goal is to get my EMR and the pharmacist's EMR exactly the same and up to date.

Physician 1206, Family Health Team, Alberta

The lack of overlap between physician and pharmacist information systems reinforces the siloed workflows of the two professions and lack of interoperability between privately-owned EMRs. However, even when pharmacists and physicians work on the same system, it can be difficult to mesh the two decision-making processes. The resulting hybrid can be inefficient, requiring back-and-forth between the patient and different HCPs.

I made some recommendations to the physician and patient, which then the physician discussed with the patient in her appointment with the patient. We also discussed, the doctor and I, after, to confirm, yes, this is what we did, and just to follow-up on the whole discussion.

Pharmacist 1124, Family Health Team, Ontario

Many participants lacked awareness of the decision-making processes of other HCPs, which left them guessing why certain decisions were made. Guesswork, thus, becomes the *de facto* process, rather than an open and collaborative process. Finally, even though Alberta pharmacists are able to prescribe and use a provincial EHR used by physicians, their experiences have been ultimately similar to the pharmacists in other provinces who did not have access to an EHR.

### Scope of Practice

Scope of practice refers to the internal and external boundaries placed on pharmacists and physicians. In many provinces, the scope of pharmacist practice has expanded to include prescribing, which has traditionally been the physician’s role. This can result in role friction.

It's been good, all the changes, for sure. […]You just learn [which physicians] who you can do it with and who you can't, and then you go with that.

Pharmacist 1114, Independent Pharmacy, Alberta

In rural areas, pharmacists have more latitude toward full scope of practice as fewer options for care are available, and they are more likely to know other local HCPs. Scarcity of services provides situations that encourage greater collaboration and partnerships due to availability as well as familiarity with colleagues.

There’s no full time physician in town…A lot of the local doctors are very open to our input and actually will seek it. Nearby doctors are a group who will cover for each other, and we know them.

Pharmacist 1110, Independent Pharmacy, Nova Scotia

Ideally, a team-based practice means that the different professions are more easily able to understand each other’s roles, including how one profession’s skills can complement another’s. Physicians generally did not consider pharmacists as partners in care and rarely mentioned active collaboration.

Yeah. Things are good with my pharmacist and I. We're still trying to work on enhancing our relationship but definitely the trust exists there and then now it's just kind of more a matter of allowing some pharmacists to feel like they can do more.

Physician 1205, Family Health Team, Ontario

Even in cases where active collaboration was spoken of in a meaningful and positive way, it was still clear that there were underlying restrictions; for example, in the above quote, while the physician spoke about collaboration, the comments qualified that only *some* pharmacists should be allowed to *feel* like they could do more. Similarly, the physician referred to the pharmacist team member as “my pharmacist,” creating in and out groups of pharmacists and reinforcing traditional power archetypes.

### Relationships Between Pharmacists and Physicians

Physician-pharmacist relationships were often influenced by physical location and institutional context. When pharmacists and physicians were colocated, particularly when a common institutional governance was present, such as a family health team in Ontario, they were able to share a common system of health records. The face-to-face interactions also allowed the pharmacists and physicians to establish personal relationships with each other. Building trusting relationships allowed for informal collaboration about patient care. Pharmacists often spoke of feeling like an outsider to care or that they were “… not wanting to bother” the physicians [Pharmacist 1107, 1108, 1109, 1121]. The limited opportunity for face-to-face collaboration artificially restricted the pharmacist’s ability to support the patient.

Pharmacists also often felt that they had to navigate the authority of physicians when assessing medication, and that, due to their perceived role in health, they were not able to influence care to the best of their abilities.

I notified a patient’s physician to a contraindicated drug given by a patient’s psychiatrist. The physician didn’t feel comfortable changing the drug, and the psychiatrist said, well, I'm not changing mine, I have him on what I want him to be on. The neurologist, I couldn't get in touch with him, and then the group home, they were almost a little bit, “we wish you hadn't put your hands in the pot, there's too many people trying to mess things up.” It was really frustrating because there's this clear thing that could cause harm to the patient, and you almost felt like you were doing more harm than good by alerting everyone to it.

Pharmacist 1102, Independent Pharmacy, Ontario

Finally, the interviews made it clear that pharmacists’ processes for working with physicians are not designed to facilitate collaboration. Rather, they may have evolved as workarounds that compensate for the strained relationship with the physician.

Most physicians do like subtle language of requesting as to, “Can you give me the thought behind prescribing this because we're just not sure, we want to make sure the patient understands it well or providing recommendations.”

Pharmacist 1116, Alberta, Chain Pharmacy

## Discussion

### Principal Findings

This project examines how physicians and pharmacists communicate patient-focused medication information with each other to inform the design of EHRs for IP-SDM. There is limited research on how EHRs currently impact IP-SDM and the potential they have for improving collaboration. We can see that the limited communication between physicians and pharmacists is strongly dependent on the relationship between them. The suboptimal management and use of medication have already been well documented, suggesting that we may not be optimally positioned to provide accessible, effective, and affordable medication management as patient need rises over the coming decade [33]. Before pharmacists and physicians can share medication-related decisions with patients, they themselves need access to comprehensive information. Furthermore, they must be prepared to share information about decision-making and to develop strategies for interprofessional collaboration that do not rely on colocation or a common institutional EMR or EHR. The findings of this study point to a status quo where integrated provider medication management and IP-SDM are an exception rather than the rule in community settings.

Workable solutions to how information is shared are both social and technical. Most electronic health information systems are capable of semantic interoperability, where a receiving information system is able to clearly interpret information in exactly the same way as the sending information system. Use of vocabularies, including RxNorm, and structured documents, such as the Clinical Document Architecture and Fast Healthcare Interoperability Resources, supports interoperability [34]. As beneficial as these may be, the competitive market forces the costs that rarely support this option, despite its popularity among providers. Despite pharmacists having played an integral role in delivering high-quality clinical care in hospitals for decades, this study highlights the slow progress toward integration and IP-SDM acceptance in the community. Our research supports the idea that social factors such as professional acceptance, institutional structures, and trusting versus nontrusting relationships are more significant barriers to the adoption of EHRs into patient care compared with technical challenges.

Kannampallil et al [35] have noted that “complex systems can appear very different, depending on the aspects, granularity, and circumstances that the researcher chooses to focus on.” By focusing on the relationship between physicians and pharmacists in this study, we saw that each health care profession has access to critical information that the other profession does not (eg, pharmacists do not have access to information about a medication’s reason for use and physicians do not have access to adherence information). These reasons are related to inadequate systems for health information exchange as well as missing professional standards that encourage comprehensive medication information exchange.

Our findings on communication, information, and process mirrored Bardet et al’s meta-model on physician and community pharmacist collaboration [36]; they identified that early on in a collaboration, key elements include trustworthiness and clarity around roles. Physicians and pharmacists also need to develop an interdependence; establish interest, skills, and positive perceptions; have clear expectations; and build a relationship that is grounded in trust [37,38]. Open and bidirectional communication is also important [36]. Our findings add to the work of Bardet et al by highlighting how the disconnected computer systems and decision processes limit collaboration between pharmacists and physicians. All participants were enthusiastic about the potential for provincial EHRs to improve information sharing and communication [39]. A well-designed EHR could also facilitate many components of a successful collaboration. Specifically, it has the potential to foster IP-SDM and level the playing field for understanding around information, process, and communication.

According to a review of IP-SDM by Dogba et al, safe and high-quality health care depends on increased levels of collaboration among HCPs and better engagement with patients [40]. In our study, all participants voiced their support for IP-SDM in general. However, when it came to giving examples, only one physician was able to describe an instance of IP-SDM in practice, and no pharmacists or physicians were able to clearly articulate a shared vision for IP-SDM. Moreover, participants had reservations about their patients’ ability to make decisions. They referenced the notion that HCP training and experience enable them to know what is “best for the patients.” Patel et al [41] have referred to this as a “cautious willingness” to participate in IP-SDM due to fears over patient competence, motivations, and dishonesty about adherence.

The notion of “cautious willingness” also applies to HCP collaboration [42]. Physicians are cautious about giving up a perceived ownership of a patient’s care, and pharmacists are equally cautious about making physicians feel like they are trying to take over the care. The reluctance of pharmacists to embrace a full scope of practice also reflects serious concerns about missing information. In the interviews, it was clear that pharmacists perceive themselves as the last gatekeeper of a patient’s well-being, yet they are unable to perform that function.

Elwyn et al [43] noted that HCPs often miss the second half of a consultation, where IP-SDM occurs. We would argue that the second half of the medication-related consultation is where IP-SDM and the pharmacist belong. Physicians have the unique expertise to focus on the diagnoses in the first half of the consultation. Pharmacists, however, have the expertise required to help the patients understand and choose a treatment option that is consistent with their needs and preferences. However, pharmacists cannot act until they have access to the right information at the right time and have a bidirectional communication with the physician. Ultimately, research should evaluate the link between all interactions in the health care process that impact patient and clinician decision-making.

### Strengths and Limitations

As part of a larger mixed methods study, the insights presented here are derived solely from the interviews of pharmacists and physicians. Although these analyses reveal perceptions about and barriers to IP-SDM and collaboration, they do not reflect a complete analysis of all data collected, specifically the data collected from patients. However, in the context of gaining a deep understanding of physician-pharmacist communications and relationships, this analysis is a critical step in building a holistic model of IP-SDM related to medication management. In addition, while the sample includes pharmacists across all 4 provinces, recruitment challenges limited the participation of physicians in each of the 4 provinces, especially in Nova Scotia. Given the similarities in policies and practice across Canadian provinces and the inclusion of a variety of physician perspectives, we believe this had little to no impact on our results. Finally, differences in interviewers’ approaches to semistructured interviews may have led to differing emphasis on IP-SDM and collaboration. While the benefit of a multidisciplinary research team is stronger objectivity stemming from a variety of research, professional, and patient backgrounds, this study might have been strengthened even more if the research team had employed prolonged engagement. Although important, due to interview time constraints, we could not explore physicians’ perceptions of pharmacists prescribing, adapting, or cancelling medications; the influence of these perceptions is suggested to be explored in future research.

### Conclusion

Our study shows that until pharmacists can see the reason for which a medication is prescribed and physicians gain insight into adherence, neither group will be fully able to work together to make medication-related decisions collaboratively. The major barriers to collaboration include poor communication systems with minimal interinstitutional information exchange, and even when an EHR exists, competing decision-making processes are most often present. We identified the potential to build EHRs that not only better facilitate access to information but also allow for processes that better accommodate collaborative care and enable better understanding of the pharmacist’s scope of practice. Future research should focus on the alignment of EHRs with interprofessional decision-making process, which can foster both intra- and interinstitutional collaboration and information sharing to best support IP-SDM.

## Appendix

Multimedia Appendix 1 Interview Guide.

## Abbreviations

ADE: adverse drug effect
EHR: electronic health record
EMR: electronic medical record
HCP: health care professional
IP-SDM: interprofessional shared decision-making
SDM: shared decision-making
SHARE: Secure Health Access Record
