# Supplementary material for: Physician and Pharmacist Medication Decision-Making in the Time of Electronic Health Records: Mixed-Methods Study
Source: JMIR Hum Factors. 2018 Sep 25;5(3):e24. doi: 10.2196/humanfactors.9891 (PMC6231837; doi:10.2196/humanfactors.9891)
Supplement: Multimedia Appendix 1 [file humanfactors_v5i3e24_app1.pdf]

Participant ID#:________________________Date:______________________

INTERVIEW QUESTIONS FOR PHYSICIANS

Decision Making:

We're going to start by talking about how you work with patients and pharmacists

1. Tell me what it's like in your clinic.

2. Think back to the last time you prescribed a medication, can you tell me about that? (*Probing questions: Describe to me how you present a patient with different treatment options? Tell me how you take into account the values of the patients? How do you decide what information to tell them? How do you evaluate their health literacy?)*

3. When looking at a medication prescribed by someone else, tell me how you find out the indication for the patient's medication?

4. Can you describe for me how you follow medication adherence in your patients? How do you find out a patient decided to stop taking medication? Or that they changed how they take a medication? When would you call a pharmacist?

5. We're going to ask you questions about pharmacists. When I say pharmacist, who comes to mind for you? What pharmacists do you work with?

6. Think back to the last time you identified a problem with a medication, can you tell me about that? If you had a question about the prescription, what would you do first? When would you call a pharmacist? *(Probing options: What do you generally need to access? Over the last week, when did you need to get in touch with a pharmacist? What have you found to be the best, or easiest way? How do you know when a pharmacist got the information you wanted to share? Do you think there's a way to make it easier? How often do you talk to a pharmacist on the phone? Through fax? When do you call them? When do you fax them?)*

7. When was the last time you disagreed with a pharmacist about a patient's treatment? How was it resolved?

Use of computer systems

Now we're going to switch over to a discussion of your computer systems.

1. What computer system do you use? *(Probing options: How long have you had it in place? Why did you choose this system? Who purchased it? Have you worked between different systems? What system do you prefer and why?)*

2. Think back to the last patient you saw. Talk me through how you used your [ ] system. *(Probing options: What information did you look at first? Where did you spend most of your time? Would you say this is the standard use of the system)*

3. Describe how easy or difficult it is to use your [ ] system to review a patient's medications? What about the medical history?

Participant ID#:________________________Date:______________________

4. Describe how <u>useful or not useful</u> your [ ] system is for reviewing a patient's medications. What about medical history?

5. What do you like most about your [ ] system? What do you like least?

6. Describe to me how you access <u>other</u> patient records like lab values, x-rays or hospital discharge summaries? What do you look for the most?

7. Tell me how you use electronic health records that are not included in your EMR? (AB: Netcare; SW Ontario: ClinicalConnect; NS: SHARE; QC: DSQ) Why do you generally need to access them?

8. Describe an ideal computer system for your clinic. Do you foresee any barriers to using even an ideal system – even in an ideal world? How would you fix those barriers?

9. Now we've talked about your practice and your computer systems. What is your take home message for us?

INTERVIEW QUESTIONS FOR PHARMACISTS

<u>Decision Making:</u>

<u>*Interviewer: Okay, we're going to start by talking about how you work with patients and physicians.*</u>

1. Tell me what it's like in your pharmacy.
2. Think back to the last time a patient asked you to help them make a choice about a medication, can you tell me about that? (*Probing questions: Describe to me how you present a patient with different treatment options? Tell me how you take into account the values of the patients? How do you decide what information to tell them? How do you evaluate their health literacy?)*
3. Tell me how you find out the indication for a patient's medication?
4. How do you follow medication adherence in your patients? How do you find out a patient decided to stop taking medication? Or that they changed how they take a medication?
5. We're going to ask you questions about physicians. When I say physician, who comes to mind for you? What physicians do you work with?
6. Think back to the last time you identified a side effect or drug interaction, can you tell me about that? If you had a question about the prescription, what would you do first? Why? Describe to me what you do when you need to get in touch with a physician? Describe to me how you present a physician with different treatment options? (*Probing options: What have you found to be the best, or easiest way? How often do you talk to a physician on the phone? Through fax? When do you call them? When do you fax them? How do you know when a physician got the information you wanted to share? Do you think there's a way to make it easier?*

<u>Computer Systems</u>

Now we're going to switch over to a discussion of your computer systems.

1. When you start your day/shift, tell me what information you look at first?
2. When you open a patient's record/medication order, tell me what you look at first?

Participant ID#:________________________Date:______________________

3. What computer system do you use? *(Probing options: How long have you had it in place? Why did you choose this system? Who purchased it? Have you worked between different systems? What system do you prefer and why?)*
4. Think back to the last patient you saw. Talk me through how you used your [   ] system. *(Probing options: What information did you look at first? Where did you spend most of your time? Would you say this is the standard use of the system?*
5. Describe how <u>easy or difficult</u> it is to use your [   ] system to review a patient's medications? What about the medical history?
6. Describe how <u>useful or not useful</u> your [   ] system is for reviewing a patient's medications. What about medical history?
7. What do you like most about your [   ] system? What do you like least?
8. Describe how you document the care you provide during dispensing. What about medication reviews?
9. In addition to the [   ] system, describe how you access <u>other</u> patient records like lab values, x-rays or hospital discharge summaries? What do you look for the most? (AB: Netcare; SW Ontario: ClinicalConnect; NS: SHARE; QC: DSQ) Why do you generally need to access the them?
10. Describe an ideal pharmacy computer system? *(Probing: Do you foresee any barriers to using even an ideal system – even in an ideal world? How would you fix those barriers.)*
11. Now that we've talked about your practice and your computer systems, what is your take home message for us?
